# Preparation and Modification of Collagen/Sodium Alginate-Based Biomedical Materials and Their Characteristics

**DOI:** 10.3390/polym16020171

**Published:** 2024-01-06

**Authors:** Leilei Sun, Yanyan Shen, Mingbo Li, Qiuting Wang, Ruimin Li, Shunmin Gong

**Affiliations:** College of Life Science, Yantai University, Yantai 264005, China; yyshenqx1999@s.ytu.edu.cn (Y.S.); mbli@ytu.edu.cn (M.L.); 1981979471@s.ytu.edu.cn (Q.W.); remmy17861135192@s.ytu.edu.cn (R.L.); g18807041394@s.ytu.edu.cn (S.G.)

**Keywords:** collagen, sodium alginate, cross-linking, biomedical material

## Abstract

(1) Background: Collagen and sodium alginate are commonly used in the field of biomedical materials due to their excellent biocompatibility. This study focuses on the preparation, modification, and characterization of collagen/sodium alginate (C/SA)-based biomedical materials. (2) Methods: The characteristics, including surface chemistry, mechanical properties, hygroscopicity, and porosity, were analyzed. The hemostatic activity in vitro was measured using a blood clotting assay and dynamic blood clotting assay. (3) Results: The results from microstructure and porosity measurement revealed that all of the sponges exhibited a porosity of more than 95 percent. The sponge cross-linked with 1-ethyl-3-(3-dimethylaminopropyl) carbodiimide/N-hydroxysuccinimide (EDC/NHS) showed better tensile strength and lower elongation at break. The sponges cross-linked with EDC/NHS and oxidized sodium alginate (OSA) exhibited the highest hygroscopicity in comparison with the uncross-linked sponge. (4) Conclusions: Our study demonstrated that the C/SA-based material we prepared exhibited a high level of porosity, enabling efficient absorption of tissue exudate and blood. Additionally, the materials revealed excellent hemocompatibility, making them suitable for use as a hemostatic dressing in the field of biomedical materials.

## 1. Introduction

The utilization of natural polymers in hemostatic and wound healing materials is a topic of significant research due to their exceptional biodegradability, biocompatibility, viscoelasticity, and ease of processing [1]. Among the natural polymers, collagen and sodium alginate (SA) are commonly studied [2]. Collagen, as the major protein of the extracellular matrix (ECM), has emerged as an effective dressing for wound hemostasis, as well as for repairing and filling defects in both hard and soft tissues [3]. It possesses excellent properties such as good biocompatibility, biodegradability, and low immunogenicity [4]. Collagen is found in different types in the different tissues, with type I collagen being the most common, abundant, and widely distributed among the 29 known types of collagen [5,6]. SA is a water-soluble linear natural polysaccharide extracted from brown algae or sargassum. It is present in kelp in concentrations of 30–40% and appears as a white or yellowish powder [7]. SA possesses several advantageous properties, including low toxicity, low immunogenicity, rapid biodegradation, and high hydrophilicity. SA possesses a negative charge that enables it to form hydrogels by cross-linking with metal ions (e.g., Ca^2+^) via carboxyl groups. This unique property enhances its suitability for use in hemostatic materials [8]. As a result, the use of SA has been widely explored in different fields, including biomedicine and tissue engineering dressings. Previous studies have demonstrated that incorporating SA into collagen-based biomaterials enhances their hemostatic properties, and researchers have also investigated the positive effects of mechanical and biochemical signals on cells, including cell viability, integrin-specific attachment, spreading, and proliferation [9,10].

Bovine and porcine sources have traditionally been used for clinical collagen-based products. However, there are concerns surrounding the use of these sources due to outbreaks of zoonotic diseases such as Bovine Spongiform Encephalopathy (BSE) and Foot and Mouth Disease (FMD), as well as religious prohibitions and animal protection concerns. These factors are threatening and restrict their use in daily life [11,12]. As a result, there has been growing interest in marine collagen, which offers advantages such as safety, reliability, and no risk of viral transmission. Recent research has shown that tilapia-derived native collagen is a promising biomaterial for healthcare applications [13,14,15]. 

The biomedical potential of natural collagen is limited due to its poor mechanical strength, thermal stability, and enzyme resistance. However, researchers have used exogenous chemical, physical, or biological cross-linking methods to modify the molecular structure of collagen, aiming to minimize degradation and enhance mechanical stability [16,17]. Various chemical cross-linkers, such as glutaraldehyde (GA) and formaldehyde, have been widely employed for cross-linking collagen-based scaffolds [18]. Although these chemical cross-linkers have been shown to improve the physicochemical properties of collagen-based scaffolds, it is crucial to take into account their potential cytotoxic effects [19]. The 1-ethyl-3-(3-dimethylaminopropyl) carbodiimide/N-hydroxysuccinimide (EDC/NHS) cross-linking method involved the activation of carboxyl groups of aspartic acid and glutamic acid, which then reacted with amine groups of lysine and hydroxylysine to form an amide bond. Additionally, it did not contribute to the cross-linking bond and acted as a zero length cross-linker. SA can also be oxidatively modified with NaIO_4_, resulting in the partial oxidation of the hydroxyl group to an aldehyde group. The resulting oxidized sodium alginate (OSA) exhibits biocompatibility and improved physical and chemical properties. The introduction of the active aldehyde group also makes it a promising biological cross-linking agent [20]. 

This study presents a green strategy for cross-linking a sponge dressing made of collagen and SA using OSA as a cross-linking agent. The aldehyde group in OSA reacts with the amine group in collagen to form a chemical cross-linking network. In addition, C/SA was also cross-linked using an EDC/NHS cross-linker. The study investigates the effects of the sponges on micromorphological stability, mechanical behavior, physicochemical properties, hemostatic activity in vitro, and biocompatibility. This research provides a new strategy for the development of future hemostatic dressings based on C/SA.

## 2. Materials and Methods

### 2.1. Materials and Reagents

Nile tilapia skin acid-soluble type I collagen was supplied by our laboratory [15].

Male Wistar rats weighing 200–220 g were obtained from Jinan Pengyue Laboratory Animal Breeding Co., Ltd., (Jinan, China). The authors complied with the relevant guidelines for animal experiments.

SA was purchased from Bright Moon Seaweed Group Co., Ltd., (Qingdao, China). EDC and NHS were purchased from Shanghai Aladdin biochemical technology Co., Ltd., (Shanghai, China). 2-(N-morpholino) ethanesulfonic acid (MES) was provided by Beijing Solarbio Science & Technology Co., Ltd., (Beijing, China). The commercial Beiling collagen sponge was acquired from Beijing Yierkang Bioengineering Co., Ltd., (Beijing, China). All reagents used, including NaCl, Na_2_HPO_4_, CaCl_2_, acetic acid, and ethanol, were of analytical grade, except for KBr (spectrum pure) and normal saline (injection grade).

### 2.2. Methods 

#### 2.2.1. Fabrication of C/SA Sponge

For the preparation of C/SA sponge, collagen was dissolved in 0.5 M acetic acid and stirred. It was then dialyzed and prepared as a 10 mg/mL solution, which was set aside. SA was also prepared as a 10 mg/mL solution in deionized water. The two solutions were co-mixed in a ratio of 5:1. SA was slowly added to the collagen solution while stirring at high speed with a magnetic stirrer. The mixture was then centrifuged at 8000 r/min at 4 °C for 8 min to remove air bubbles. Approximately 10 mL of the pre-cursor solution was transferred to 6-well plates and pre-frozen at −40 °C. Subsequently, it was lyophilized under a vacuum of 0.06 mbar to obtain C/SA sponge [21]. Finally, the C/SA scaffolds were stored in a dryer until used for subsequent processing.

#### 2.2.2. EDC/NHS Cross-Linking of C/SA Sponge

**Figure 1:**
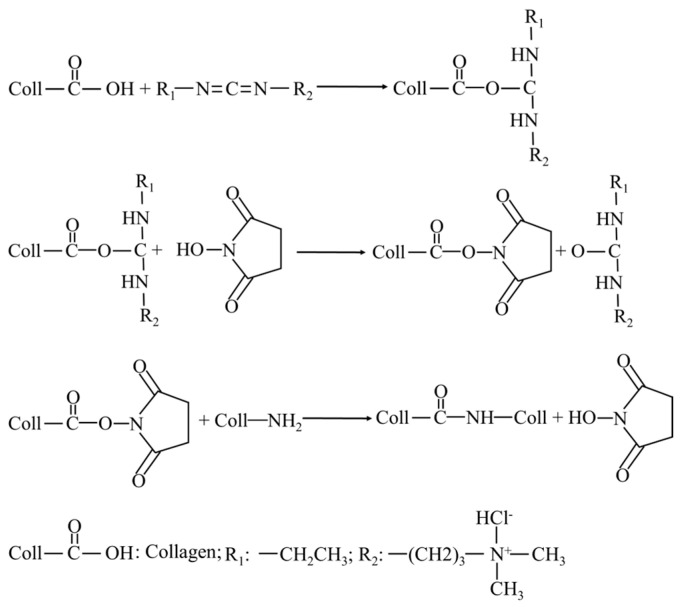
Schematic for the formation of cross-linking of EDC/NHS.

C/SA sponges were immersed in 40% ethanol containing 50 mM MES buffer (pH 5.5) for a minimum of 30 min. Then, they were immersed in a solution of 40% ethanol containing 50 mM MES buffer, along with concentrations of 20, 40, 60, 80, and 100 mM EDC and NHS (in a ratio of n_EDC_:n_NHS_ = 2.5:1) (pH 5.5). The cross-linking process was carried out at 25 °C for 4 h. Subsequently, the samples were washed twice with 0.1 M Na_2_HPO_4_, followed by washes with 1, 2, and 4 M NaCl for 4 h, overnight [22]. The washes were then repeated with deionized water and, finally, the samples were lyophilized. The schematic for the formation of cross-linking of EDC/NHS is shown in Figure 1.

#### 2.2.3. Fabrication of OSA and C/OSA Sponge

For the OSA preparation, 10 g of SA was dispersed in 50 mL of anhydrous ethanol to form solution 1. In another container, 10 g of sodium periodate was dissolved in 125 mL of water to form solution 2. Solution 2 was then added to solution 1 and the mixture was oxidized by magnetic stirring at room temperature, away from light, for 8 h. After that, an amount of ethylene glycol equal to the moles of sodium periodate was added, and the oxidation reaction was terminated for 30 min. The resulting reaction mixture was poured into a large amount of anhydrous ethanol (V_reaction mixture_:V_anhydrous ethanol_ = 1:5) with vigorous stirring to precipitate the product. After filtration, the precipitate was dissolved in deionized water, and then precipitated again with anhydrous ethanol. The precipitate was dissolved in deionized water and dialyzed for 3 d to remove any unreacted small molecule impurities, such as sodium periodate and glycol. The remaining liquid in the dialysis bag was precipitated once more with anhydrous ethanol and filtered to obtain a white product [23]. Finally, the product was placed into a surface dish and lyophilized to obtain sodium alginate oxide, which could be used for subsequent cross-linking.

**Figure 2:**
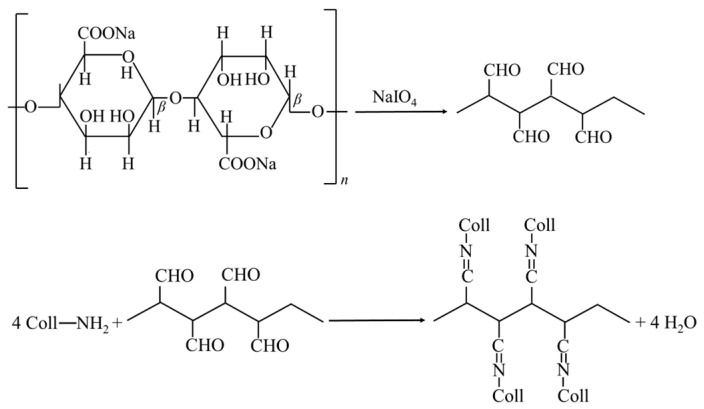
Schematic for the formation of cross-linking of OSA.

For the preparation of C/OSA sponge, collagen was dissolved in 0.5 M acetic acid and stirred. The solution was then dialyzed and prepared into a 10 mg/mL solution for future use. OSA was separately prepared as a 10 mg/mL aqueous solution. The collagen and OSA were co-mixed in a ratio of 5:1. The OSA solution was slowly added to the collagen solution while stirring at high speed with a magnetic stirrer. The cross-linking process was carried out at 4 °C for 24 h and then subjected to centrifugation at 8000 r/min at 4 °C for 8 min to remove any foam. Approximately 10 mL of the precursor solution was transferred to 6-well plates and pre-frozen at −40 °C. Subsequently, it was lyophilized under a vacuum of 0.06 mbar to obtain C/OSA sponge. The schematic for the formation of cross-linking of OSA is shown in Figure 2.

#### 2.2.4. Microstructure 

The positive and inverse surface, cross section, and longitudinal section of each C/SA-based sponge sample was subjected to gold sputtering with an ion coater (IB 3, Eiko, Tokyo, Japan). The resulting images were observed at 100× magnification using a scanning electron microscopy (JSM-840, JEOL, Tokyo, Japan) at a voltage of 5 kV.

#### 2.2.5. Fourier Transforms Infrared Spectroscopy (FTIR) 

The chemical structure of the materials was analyzed using an infrared spectrophotometer (iS 10, Thermo-Nicolet Co., Waltham, MA, USA). Briefly, the samples were mixed with KBr in a ratio of approximately 1:100 (*w*/*w*) and compressed into tablets. The data acquisition rate was set at 2 cm^−1^ with a scanning range of 4000–400 cm^−1^. The peak wavelength values were processed and analyzed using OMNIC 8.0 software.

#### 2.2.6. Mechanical Properties 

The mechanical tensile strength and elongation at break of C/SA-based sponge samples were evaluated using a texture analyzer (TMS-Pro, Food Technology Corporation, Sterling, VA, USA), following the guidelines of GB/T 1040.3-2006 [24]. The sponge samples were cut into dimensions of 25 mm × 10 mm, and the ends were securely fastened to the pulling force machine with an initial distance of 15 mm. A total of 9 samples were taken for each series to determine the tensile strength and elongation at break, which were calculated using the following formula:

(1) $$TS=\frac{F}{S}\times 1000$$

(2) $$E(\%)=\frac{∆L}{L}\times 100$$

where TS represents tensile strength (KPa); *F* represents the maximum tension when the sample was broken (N); *S* represents cross sectional area of the sample (mm^2^); E represents elongation at break (%); Δ*L* represents extended displacement when the sample was broken (mm); and *L* represents gauge length (mm).

#### 2.2.7. Hygroscopicity 

The determination of hygroscopicity was conducted according to YY/T 0471.1-2004 [25], with slight modifications. The samples, measuring 1 × 1 cm, were precisely weighed (*W*_d_) and immersed in a pre-heated aqueous brine solution at 37 °C. This solution contained 142 mM Na^+^ and 2.5 mM Ca^2+^, which mimicked the components found in human serum and wound exudate as per EN 13726-1 [26]. The samples were then incubated in a 37 °C water bath for 30 min. Afterward, one end of the sponge was removed from the liquid using a blunt tweezer, suspended for 30 s to eliminate surficial water, and then accurately weighed (*W_w_*). All obtained hygroscopicity data were calculated as follows:

(3) $$Hygroscopicity=\left(W_{w}-W_{d}\right)/W_{d}$$

#### 2.2.8. Porosity Measurement 

The porosity of the sponge samples was determined using the liquid replacement method [27]. Anhydrous ethanol was used as the replacement liquid. Porosity was determined by observing the ability of absolute ethanol to penetrate the sponge pores without causing any shrinkage or swelling. The procedure involved immersing the sample sponges in a measuring cylinder filled with a known volume of ethanol (*V*_1_). Ultrasonic degassing was performed until the sponge pores were saturated with ethanol. The total volume of ethanol and ethanol-immersed sponges was then recorded (*V*_2_). The sponge was quickly removed using forceps, and the volume of ethanol remaining in the cylinder was recorded (*V*_3_). The porosity of the sponge was calculated using the following formula:

(4) $$Porosity(\%)=V_{P}/V_{t}\times 100=\left(V_{1}-V_{3}\right)/(V_{2}-V_{3})\times 100$$

where *V*_p_ and *V*_t_ are the pores volume of sponges, and the total volume of sponges, respectively.

#### 2.2.9. Hemostatic Activity In Vitro 

##### Blood Clotting Assay In Vitro

All C/SA-based sponges were cut into pieces measuring 5 mm in length and 5 mm in thickness. An in vitro coagulation assay was performed in tubes. Each tube (n = 3 for each sponge) was filled with 100 µL of saline, and the sponges were immersed in the tubes for 5 min at 37 °C. Subsequently, 500 µL of freshly collected blood samples from male Wistar rats were added to each test tube. A timer was started as soon as blood began to flow into the tube. The test tubes were tilted once every 20 s until a thrombus formed. After tilting, the next test tube was started, and the time at which the blood in the last test tube coagulated was recorded as the blood coagulation time.

##### Dynamic Blood Clotting Assay In Vitro

C/SA-based sponge samples measuring 1 × 1 cm were placed in glass dishes and preheated at 37 °C for 5 min. Then, 250 μL of citrated whole blood obtained from Wistar rats in a ratio of 9:1 was slowly dispensed onto the surface of the samples. Subsequently, 20 μL of 0.2 M CaCl_2_ solution was added. The sponges were then incubated at 37 °C for 1, 3, 7, 15, and 30 min. After that, 50 mL of distilled water was carefully added to the glass dishes and shaken at 80 rpm for 10 min. The absorbance of the resulting hemoglobin solution was recorded at 540 nm (Abs of sample). Furthermore, the absorbance of 250 μL of anticoagulant blood added to 50 mL of distilled water, used as a reference (Abs of blank), was assumed to be 100 as a control value. The blood clotting index (BCI) of C/SA-based sponges was calculated using the following equation:

(5) $$BCI(\%)=({OD}_{s}/{OD}_{b})\times 100$$

where *OD*_s_ and *OD*_b_ are the absorbance of samples and control, respectively.

#### 2.2.10. Hemolysis Assay 

To determine the hemocompatibility of C/SA-based dressings, we slightly modified the hemolysis test proposed by the American Society for Testing and Materials (ASTM F 756-00, 2000) [28]. Prior to exposing the samples to blood, each C/SA-based sponge sample (1 cm^2^) was immersed in a test tube containing 10 mL of normal saline at 37 °C for 30 min. Afterward, the saline was discarded and the samples were immersed in 0.2 mL of diluted anticoagulated whole blood from Wistar rats. The blood was diluted with saline at a ratio of 4:5 (*v*/*v*) and kept at 37 °C for 60 min. Negative (0% hemolysis) and positive (100% hemolysis) controls were established by adding 0.2 mL of diluted blood to 10 mL of deionized water and 10 mL of saline, respectively. The amount of released hemoglobin was determined using a spectrophotometer (Metash, Shanghai, China) at 540 nm. The blood hemolysis experiments were replicated three times under identical conditions. The obtained hemolysis ratio (HR) was evaluated as a percentage of complete hemolysis, calculated as follows:

(6) $$HR(\%)=\left(A_{s}-A_{n}\right)/(A_{P}-A_{n})\times 100$$

where *A*_s_, *A*_n_, and *A*_p_ are the absorbance of sample, negative control, and positive control, respectively.

#### 2.2.11. Statistical Analysis 

All statistical data were analyzed using SPSS19.0 software. The data were presented as means ± standard deviations. Statistical comparisons were performed using one-way analysis of variance (ANOVA) with post hoc tests and students’ *t*-tests. A *p*-value of less than 0.05 was considered statistically significant.

## 3. Results and Discussion

### 3.1. Morphology of C/SA-Based Sponges 

**Figure 3:**
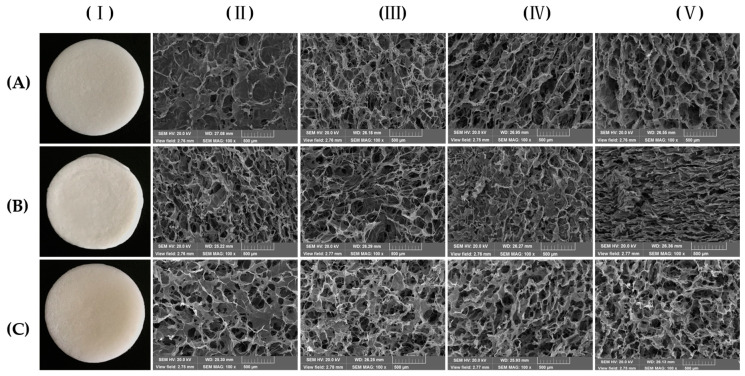
Macro-morphology (**I**) and corresponding SEM micrographs for positive surface (**II**), inverse surface (**III**), cross section (**IV**), and longitudinal section (**V**) of (**A**) C/SA; (**B**) EDC/NHC cross-linked; (**C**) C/OSA sponges.

The macro-morphology and micro-morphology for the positive and inverse surface, cross section, and longitudinal section of C/SA-based sponges are illustrated in Figure 3 to compare the impact of different types of chemical cross-linking on the sponge structure. This parameter was considered significant for biomaterials. From a macro-morphology perspective, the composite materials exhibited a three-dimensional network structure. When collagen was combined with OSA, the material surface appeared yellowish. From a micro-morphology perspective, both the cross-linked sponges and the unmodified sponge exhibited a similar structure, characterized by a large number of irregular pores that were conducive to cell growth. The loose honeycomb porous structure of the sponge plays a crucial role in achieving rapid hemostasis when used as a hemostatic sponge. This structure enables the sponge to quickly absorb a significant amount of wound secretion, thereby increasing the plasma concentration at the wound site [29]. Additionally, these findings align well with the determination of porosity, indicating that the sponge promotes oxygen replenishment and cell proliferation at the wound site. Furthermore, the appropriate size of the C/SA-based sponges enhance platelet adhesion and aggregation.

### 3.2. FTIR Analysis 

**Figure 4:**
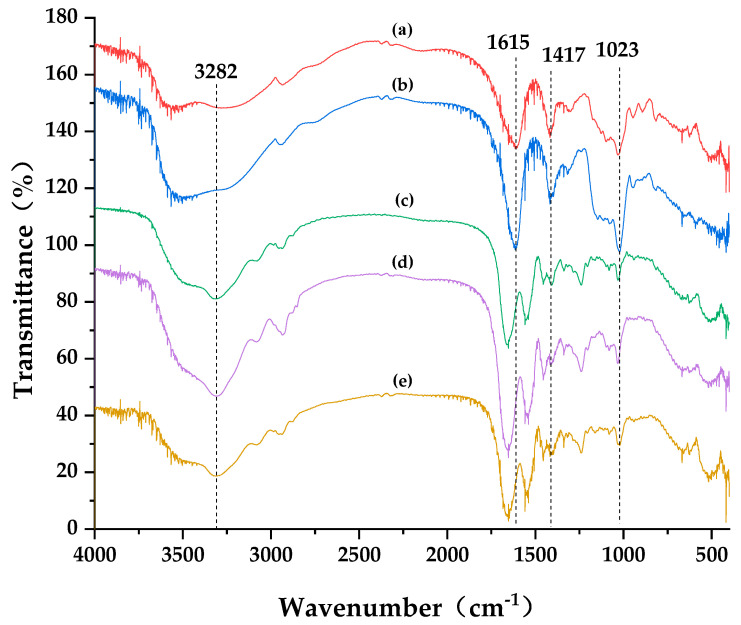
FTIR spectra of (**a**) SA; (**b**) OSA; (**c**) C/SA sponge; (**d**) EDC/NHC cross-linked sponge; (**e**) C/OSA sponge.

To analyze the changes of C/SA at the molecular level after cross-linking with EDC/NHS and OSA, FTIR spectra were collected (Figure 4). The characteristic absorption bands, including amide A, B, I, II, and III, were present in all samples. The amide A band at 3400–3440 cm^−1^ corresponds to NH-free stretching vibration. When the NH group is involved in a hydrogen bond, it shifts to a lower frequency. Cross-linking with EDC/NHS and OSA resulted in a shift of the amide A peak from 3315 to approximately 3282 cm^−1^. The stretching vibrational peaks of the O–H and N–H bonds were significantly enhanced, which can be attributed to the hydrogen bonding interactions between C/SA and collagen with EDC/NHS and OSA, respectively. This further supported the occurrence of cross-linking reactions. Moreover, a more pronounced effect was observed when cross-linked with EDC/NHS. Similar results were reported for the cross-linking of kaolin (K) into carboxymethyl chitosan (CMCS)/SA and collagen from a bovine Achilles tendon, using oxidized multi-walled carbon nanotubes with riboflavin sodium phosphate [30,31]. The presence of proteinaceous components in (c), (d), and (e) is evident from the FTIR spectra, where the bands at 1653 cm^−1^ and 1558 cm^−1^ can be assigned to amide I and II, respectively [32]. In contrast to (a) and (b), a redshift of amide II was also observed in (c), (d), and (e), which was associated with N–H bending, coupled with C–N stretching vibrations. Spectrum (b) exhibited the characteristic vibrational peak of the aldehyde group at 1615 cm^−1^, while in (e), the absorption peak of the Schiff base structure appeared at 1653 cm^−1^. The absorption peak at 1417 cm^−1^ disappeared, while two new absorption peaks appeared at 1445 cm^−1^ and 1409 cm^−1^, indicating the successful introduction of the carboxyl group. These parameters are closely related to the mechanical properties.

### 3.3. Influence of Different Cross-Linking Methods on Mechanical Properties and Hygroscopicity of C/SA-Based Sponges 

Mechanical properties are crucial parameters when considering biomedical hemostatic scaffolds [33]. Table 1 presents the comparison of tensile strength among different cross-linking methods used in the production of sponges. The cross-linked C/SA sponge, achieved through EDC/NHS, exhibited a higher tensile strength of 310.22 ± 61.96 Kpa when compared to the unmodified C/SA and C/OSA sponges.

Additionally, the evaluation of biomaterials in terms of their interaction with physiological fluids within the body takes into account the parameter of hygroscopicity. Hygroscopicity plays a significant role in cell adhesion, growth, behavior, and differentiation. Hemostatic materials with higher hygroscopicity aid in concentrating clotting factors and enhancing the rate of hemostasis. In contrast, non-cross-linked C/SA material became loose after prolonged immersion in liquid, making it difficult to accurately determine its hygroscopicity. However, sponges treated with EDC/NHS and OSA displayed good solution stability and hygroscopicity, which facilitated the absorption of wound exudate. These findings suggested that proper cross-linking could substantially improve the mechanical strength and hygroscopicity of biomaterials.

### 3.4. Porosity

Higher porosity is favorable for the hemostatic material to absorb exudate from the wound and facilitate gas exchange [34]. As depicted in Table 2, all porosities exceeded 95%. The C/SA sponge porosity was 96.87%, and the sponge porosities after cross-linking with EDC/NHS and OSA were 96.78% and 96.97%, respectively. The results showed that the addition of cross-linking treatment or compounding collagen with oxidized sodium alginate did not have a statistically significant effect on sponge collagen porosity. Therefore, the three prepared C/SA-based sponges can enhance cell proliferation, provide subcutaneous tissues with oxygen, eliminate necessary gases for growth, and maintain appropriate environmental humidity. These factors facilitate the growth of epidermal tissues, which is a crucial requirement for the preparation of tissue engineering scaffolds.

### 3.5. Hemostatic Activity In Vitro

#### 3.5.1. Clotting Capability

Clotting time is a direct method used to assess the hemostatic capacity of biomedical materials. In this study, coagulation experiments were performed using fresh male Wistar rats’ blood, and the response of the implanted device was evaluated by simulating it in a test tube. The clotting time of the blank control, C/SA-based sponges, and a commercially available sponge are compared and presented in Table 3. The results demonstrated that our fabricated C/SA-based sponges significantly shortened the hemostatic time compared to the control (161 ± 8 s, *p* < 0.05). Additionally, the chemical cross-linking did not noticeably affect the clotting capability of the C/SA-based sponges. Previous studies have reported that collagen sponges can activate platelets, and the introduction of SA can further enhance blood coagulation [21,35]. Therefore, our fabricated materials offer a fast and effective solution for hemorrhage control.

#### 3.5.2. Whole Blood Clotting in Vitro

**Figure 5:**
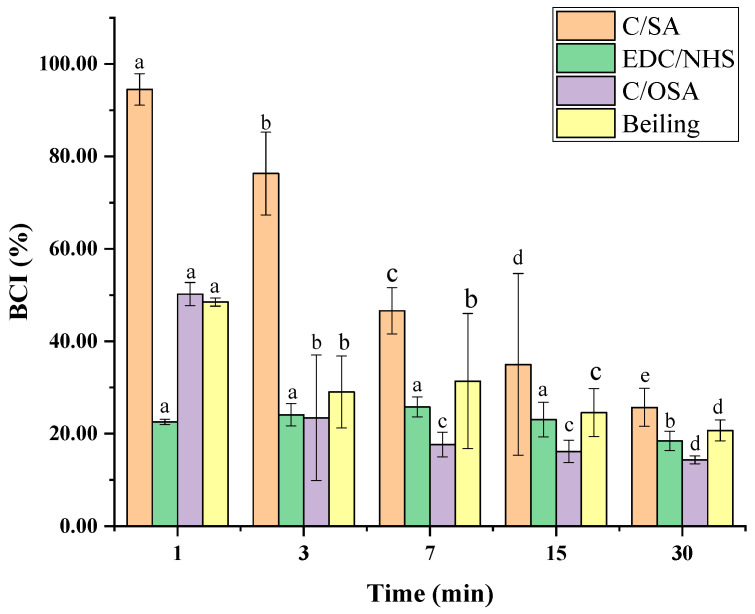
The blood clotting kinetics of C/SA-based sponges. Different lowercase letters in the same cross-linking group represent significant differences (*p* < 0.05).

The interaction of hemostatic dressings with plasma proteins can potentially affect protein function. Therefore, the whole blood clotting assay in vitro is used to assess whether medical hemostatic dressings interfere with normal plasma coagulation. In vitro evaluation of the hemostatic ability of the sponges was conducted using BCI. A lower BCI indicates a stronger hemostatic potential of the materials [36]. Anticoagulant whole blood was applied in the assay to magnify the differences in clotting ability among various hemostatic dressings. The results of blood clotting kinetics are illustrated in Figure 5. All of the sponges exhibited coagulated activity. EDC/NHS cross-linked and C/OSA sponges showed better coagulation activity compared to non-cross-linked C/SA sponge. The non-cross-linked C/SA sponge promoted blood coagulation within the selected time intervals, with a sharp decrease in BCI at 7 min. Meanwhile, the BCI values of EDC/NHS cross-linked, C/OSA, and commercial sponges were 22.57 ± 0.59%, 50.21 ± 2.51%, and 48.49 ± 0.89%, respectively, which were < 50% at the start of coagulation at 1 min. After an extended period of time, anticoagulant whole blood in all three samples coagulated gradually to form blood clots and the BCI value decreased to < 30% after 30 min. However, the BCI value of the EDC/NHS cross-linked and C/OSA sponges was significantly lower, especially at the start of coagulation at 1 min, indicating a faster and better blood clotting ability compared to the commercial product. The cross-linking of the C/SA sponge greatly improved its blood clotting ability, consistent with the result of water absorption capacity. The interaction between the hemostatic dressing with platelets, erythrocytes, and fibronectin in the clot may cause a modification in protein function, resulting in rapid coagulation [37,38]. In vitro experiments using whole blood clotting assay revealed that all samples exhibited a blood clotting ability, indicating the potential of the produced C/SA-based sponges as effective hemostatic dressings.

### 3.6. Blood Compatibility Evaluation

Blood compatibility is a crucial characteristic in biomedical materials [39]. To evaluate the hemocompatibility of C/SA-based sponges, we investigated their potential to induce hemolysis of red blood cells (RBCs). The blood compatibility of biomedical materials was assessed through an in vitro hemolysis assay. The extent of RBCs rupture, known as HR, indicates the release of hemoglobin when biomedical materials come into contact with blood. A lower HR value signifies better blood compatibility of the biomaterials. According to ASTM F 756-00, 2000 [28], an HR value below 5% is considered acceptable. Table 4 shows that the HR values of all of our fabricated sponges, both before and after cross-linking, were within the acceptable limits. These results demonstrated that the fabricated sponges, whether cross-linked or not, could be regarded as nearly non-hemolytic biomaterials with favorable blood compatibility.

## 4. Conclusions

In this study, composite medical materials based on C/SA were prepared using a vacuum freeze-drying method. Different cross-linking methods were employed to enhance the physicochemical and biological properties of C/SA-based sponges. The strong interactions, such as hydrogen bonding between collagen, SA, and EDC/NHS, contributed to the uniform and dense cross sectional morphology of the C/SA-based sponges. Furthermore, these interactions improved the mechanical properties, flexibility, and hygroscopicity of the sponges, compared to the non-cross-linked C/SA sponge. The sponge cross-linked by EDC/NHS exhibited superior mechanical properties, moisture absorption, and hemostatic activity in vitro, outperforming even commercial product. Notably, there was no significant change in the porosity of the sponges before and after cross-linking. Overall, this study demonstrates the potential of C/SA-based sponges as novel and safe materials for biomedical applications.

## Figures and Tables

**Table 1:** Mechanical properties and hygroscopicity of C/SA-based sponges.

	Tensile Strength (KPa) ^A^	Elongation at Break (%) ^A^	Hygroscopicity ^B^
C/SA	50.92 ± 1.84 ^a^	22.16 ± 0.99 ^a^	20.94 ± 1.66 ^a^
EDC/NHS cross-linked	310.22 ± 61.96 ^b^	20.97 ± 0.65 ^b^	25.93 ± 0.56 ^b^
C/OSA	54.44 ± 2.95 ^a^	22.14 ± 1.06 ^a^	25.12 ± 0.43 ^b^

Note: The different small letters for data in the same group represent significant difference (*p* < 0.05). ^A^ The values represent mean ± SD (n = 9). ^B^ The values represent mean ± SD (n = 3).

**Table 2:** Porosity of non-cross-linked and cross-linked C/SA-based sponges.

	C/SA	EDC/NHC Cross-Linked	C/OSA
Porosity (%)	96.87 ± 0.11	96.78 ± 0.78	96.97 ± 0.27

**Table 3:** Blood coagulation time of non-cross-linked and cross-linked C/SA-based sponges.

	Blood Coagulation Time (s)
Control	161 ± 8 ^a^
C/SA	101 ± 9 ^b^
EDC/NHS cross-linked	112 ± 6 ^b^
C/OSA	112 ± 7 ^b^
Beiling	114 ± 6 ^b^

Note: The different small letters represent significant difference (*p* < 0.05). The values represent mean ± SD (n = 3).

**Table 4:** Hemolysis ratio of C/SA-based sponges.

	Hemolysis Ratio (%)
C/SA	1.88 ± 1.67 ^a^
EDC/NHS cross-linked	1.53 ± 2.65 ^b^
C/OSA	1.79 ± 2.35 ^a^
Beiling	4.92 ± 1.63 ^c^

Note: The different small letters represent significant difference (*p* < 0.05). The values represent mean ± SD (n = 3).

## Data Availability

The datasets generated and analyzed during the current study are available from the corresponding authors upon reasonable request.
